# Tinzaparin for Long-Term Treatment of Venous Thromboembolism in Patients With Cancer: A Systematic Review and Meta-Analysis

**DOI:** 10.1177/1076029617696581

**Published:** 2017-03-14

**Authors:** M. José Martínez-Zapata, Alexander G. Mathioudakis, Shaker A. Mousa, Rupert Bauersachs

**Affiliations:** 1 Public Health and Clinical Epidemiology Service, Instituto de Investigación Biomédica Sant Pau, CIBERESP, Barcelona, Spain; 2 Division of Infection, Immunity and Respiratory Medicine, University Hospital of South Manchester NHS Foundation Trust, University of Manchester, Manchester, United Kingdom; 3 The Pharmaceutical Research Institute, Albany College of Pharmacy and Health Sciences, Rensselaer, NY, USA; 4 Department of Vascular Medicine, Klinikum Darmstadt GmbH, Darmstadt, Germany; 5 Center for Thrombosis and Hemostasis, University Medical Center Mainz, Germany

**Keywords:** anticoagulant, tinzaparin, venous thromboembolism, malignancy

## Abstract

Patients with cancer are at increased risk of recurrent venous thromboembolism (VTE) and bleeding. Thus, long-term treatment with anticoagulants for secondary prevention is challenging. The objective of this review was to evaluate current evidence on the safety and efficacy of tinzaparin compared with other anticoagulants for long-term VTE treatment in patients with cancer. Based on a preregistered protocol, we identified randomized controlled trials (RCTs) comparing long-term tinzaparin (therapeutic dose: 175 IU/kg) versus other anticoagulants for at least 3 months after an acute episode of VTE that included adult patients with underlying malignancy. We extracted predefined, clinically relevant outcomes of patients with cancer and, using standard methodology, pooled available data and assessed risk of bias and quality of evidence for each study. Three open-label RCTs evaluating 1169 patients with cancer were included in the analysis. Tinzaparin was associated with a significantly lower risk of recurrent VTE at the end of treatment (relative risk [RR], [95% confidence interval] 0.67 [0.46-0.99]) and at longest follow-up (RR: 0.58 [0.39-0.88]) and showed a lower risk of clinically relevant non-major bleeding at the end of treatment (RR: 0.71 [0.51-1.00]). No significant between-treatment differences were found for all-cause mortality (RR: 1.09 [0.91-1.30]) or fatal and non-fatal major bleeding events (RR: 1.06 [0.56-1.99]). The overall quality of evidence was deemed moderate, mainly due to small sample size in 2 of the studies and limited number of events in the meta-analyses. In conclusion, both short- and long-term treatments with tinzaparin were found to be superior to vitamin K antagonists for avoiding recurrences of VTE.

## Introduction

Hemostasis and malignancy are strongly related,^1^ and patients with cancer are at increased risk of venous thromboembolism (VTE).^2^ Venous thromboembolism occurs 4 times more often in patients with cancer compared with the general population.^3^ However, there is a wide variability related to the cancer type and time since diagnosis.^3^ Multiple factors have been reported to increase the risk of venous thrombosis in patients with cancer. Some of these factors include chemotherapy, use of erythropoietin agents, and use of certain anticancer therapies such as thalidomide, high-dose steroids, and antiangiogenic therapy. In addition, the risk of VTE is higher in patients with coexisting chronic medical illnesses.^4^

The risk of recurrent VTE seems to be higher in patients with metastatic versus localized malignancy.^5^ Furthermore, it has been reported that the risk of recurrence is increased by factors such as interim hospitalizations, central venous catheter, and respiratory infection.^6^

A recently updated Cochrane review shows that primary thromboprophylaxis with low-molecular-weight heparin (LMWH) significantly reduces the incidence of symptomatic VTE in ambulatory patients with cancer treated with chemotherapy.^7^ Another recently published Cochrane review shows that LMWH for secondary prophylaxis, compared with vitamin K antagonists, reduces recurrent VTE events but not mortality.^8^

Tinzaparin sodium (tinzaparin) is an LMWH produced by the enzymatic degradation of porcine-derived unfractionated heparin.^9^ Tinzaparin acts as an anticoagulant by enhancing the inhibition of the activating effect of antithrombin on coagulation factors, especially Factors Xa and IIa. The ratio of anti-Xa/anti-IIa activity for tinzaparin is between 1.5 and 2.5 times the normal ratio. Subcutaneous tinzaparin increases anti-Xa and anti-IIa activities in the plasma in a dose-dependent fashion and stimulates the release of tissue factor pathway inhibitor, which contributes to its anticoagulant and potential anticancer effects;^9,10^ this could be advantageous for reducing risk of recurrence of VTE in patients with cancer.

A meta-analysis of 5 randomized controlled trials (RCTs) in patients with and without cancer found that tinzaparin may be a valuable option for long-term VTE treatment in those who have a contraindication for vitamin K antagonists or when monitoring is difficult.^11^ The meta-analysis showed no difference in symptomatic VTE after treatment but did show superiority of tinzaparin over vitamin K antagonists with regards to recurrence in patients with cancer at 1 year of follow-up.

Our objective in this systematic review is to provide an update regarding the clinical efficacy and safety and potential side effects of tinzaparin for the treatment of VTE in patients with cancer.

## Methods

This systematic review was based in a study protocol that was prospectively registered in PROSPERO database (register number CRD42016036024; available from http://www.crd.york.ac.uk/PROSPERO/display_record.asp?ID=CRD42016036024). The report was developed following guidance from PRISMA statement.^12^

### Eligibility Criteria

We included RCTs comparing head-to-head long-term (>3 months) tinzaparin (therapeutic dose 175 IU/kg; subcutaneous injection once daily) versus oral anticoagulants or any other heparin after an episode of acute deep vein thrombosis (DVT) or pulmonary embolism (PE), including adult patients with underlying malignancy of any type. Also included were RCTs evaluating non-selected adult patients, provided they included a well-defined subgroup of patients with cancer, and it was possible to identify the relevant outcomes for this subgroup of participants. A diagnosis of all episodes of thrombosis, confirmed objectively, using standard imaging techniques was a prerequisite.

### Search Sources

The electronic databases of Medline OVID (1946 to January 25, 2016), EMBASE (from 1980 to January 25, 2016), LILACS (1982 to February 10, 2016), and Cochrane CENTRAL (The Cochrane Library 2016, Issue 1) were systematically reviewed from inception to identify eligible studies. The search keywords were “tinzaparin [tiab]” OR “innohep [tiab]” OR “logiparin [tiab]” OR “tinzaparin” [Supplementary Concept].

Trial registries were also searched via the World Health Organization International Clinical Trials Platform Search Portal to identify further ongoing or completed trials. When required, the authors of the included studies were contacted in order to obtain further details. Finally, the reference lists of all trials and identifiers were also assessed.

### Outcome Measures

The primary outcome was the number of patients with at least 1 recurrent VTE event (composite of DVT and PE; incidental and symptomatic [including fatal]) at the end of the treatment period. Secondary outcomes included safety outcomes (all adverse events [AEs], all AEs related to the interventions tested, all-cause mortality at the end of treatment period and at any follow-up, major bleeding [fatal and non-fatal; defined according to International Society on Thrombosis and Haemostasis criteria]^13^ at the end of the treatment period and at any follow-up, minor bleeding [all bleedings not classified as major], clinically relevant non-major bleeding [all non-major bleedings requiring a medical or surgical intervention], and trivial bleeding [those not requiring medical or surgical intervention]); recurrent VTE at any follow-up; recurrent symptomatic DVT at the end of treatment period and at any follow-up; recurrent incidental DVT at the end of treatment period and at any follow-up; and recurrent incidental PE at the end of treatment period and at any follow-up.

### Study Selection and Data Extraction

All studies identified by the search strategies were independently assessed for inclusion by 2 review authors (M.M.Z. and A.G.M.). Data were also independently extracted by 2 review authors, using a prespecified standardized form (M.M.Z. and A.G.M.). Disagreement in study selection or extraction was resolved through discussion and consensus.

### Assessment of Risk of Bias

Two authors independently assessed the risk of bias for each included trial, in accordance with Cochrane’s Handbook.^14^ We assessed generation sequence, allocation concealment, blinding of patients and investigators, blinding of outcome assessors, incomplete outcome data, and selective reporting. Risk of bias for each of these domains was rated as low, high, or unclear. The overall risk was considered “high” if any of the domains were deemed high risk, “unclear” if any of the domains were deemed unclear risk plus none of high risk, and “low” if all domains were deemed low risk.

We had planned to explore whether the review was subject to publication bias by means of a funnel plot. However, we could not conduct this analysis because the number of included studies was less than 10.^15^

### Assessment of Heterogeneity

We used the *I* ^2^ statistic to measure statistical heterogeneity between trials in each analysis; this describes the percentage of total variation across trials, which is due to heterogeneity rather than to sampling error.^16^ We considered a substantial statistical heterogeneity if the *I* ^2^ was greater than 75%.^14^ If substantial heterogeneity was detected, we explored its sources by prespecified subgroup analyses.

### Data Synthesis

The effect of treatment with tinzaparin was estimated with pooled relative risks (RRs) and their corresponding 95% confidence intervals (CIs). Pooled estimates were computed with the Mantel-Haenszel method, under a random-effects model.^17^

Analysis of the primary outcome, all recurrent VTE, was stratified by the type of anticoagulants; analysis of the secondary outcome, non-major bleeding, was also stratified (eg, minor bleeding, clinically relevant non-major bleeding, and trivial bleeding) due to different definitions used in the literature, and the overall results were not pooled.

We used Review Manager Software (RevMan 5, Cochrane Community) to perform all statistical analyses.

### Subgroup Analysis and Investigation of Heterogeneity

Subgroup analyses were restricted to the review’s primary outcome. Three subgroup analyses were performed: by the type of oral anticoagulant; by the duration of treatment at 3, 6, and ≥12 months; and by the length of follow-up at 3, 6, and ≥12 months.

### Sensitivity Analysis

We planned to conduct a sensitivity analysis excluding studies at high risk of bias; however, this was not possible as all trials were at high risk of bias due to the open-label study design. We did perform a sensitivity analysis including only patients who complied with the protocol of the included studies.

### Quality of Evidence

We used GRADE methodology^18^ to assess the quality of the body of evidence for the outcomes: all recurrent VTE, all-cause mortality, major (fatal and non-fatal) bleeding, clinically relevant non-major bleeding, recurrent symptomatic DVT, and recurrent (fatal and non-fatal) symptomatic PE. This approach assessed the quality of the body of evidence per comparison and outcome, taking into account the risk of bias across included studies, indirectness, inconsistency, imprecision, and the publication bias. The GRADE Working Group classifies evidence in 4 grades—(1) high quality: further research is very unlikely to change our confidence in the estimate of effect; (2) moderate quality: further research is likely to have an important impact on our confidence in the estimate of effect and may change the estimate; (3) low quality: further research is very likely to have an important impact on our confidence in the estimate of effect and is likely to change the estimate; (4) very low quality: there are many uncertainties about the estimate.

We present the outcomes and quality assessments in table format, which was constructed using the GRADEPro software version 3.0 (https://gradepro.org/).

## Results

**Figure 1.**
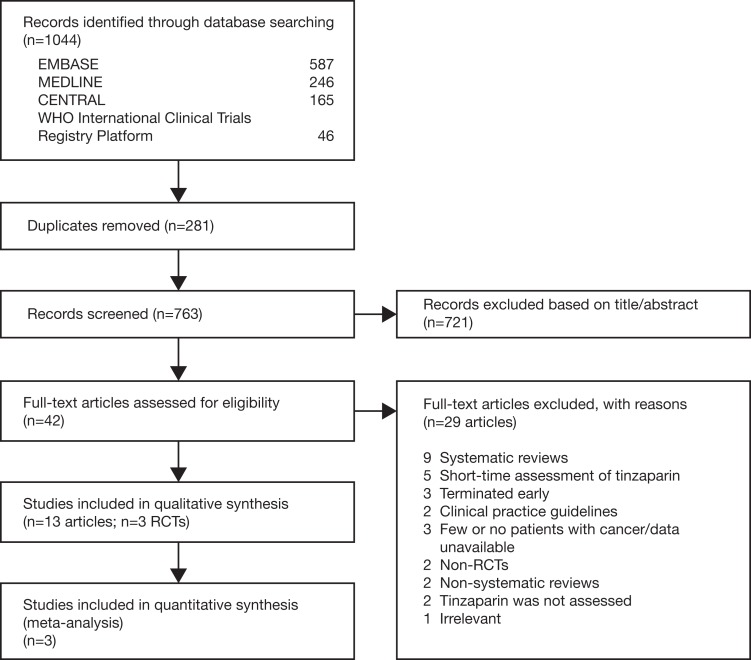
PRISMA flow diagram.

The search strategy identified 1044 relevant references. From these, we retained 763 records after duplicates were removed. Three trials met the inclusion criteria and were reported in 13 articles (Figure 1).^19–21^

### Included Studies

We included 3 RCTs.^19–21^ One RCT (the CATCH trial)^19^ was specific for patients with cancer, but the other 2 studies (the LITE trial and the Romera trial)^20,21^ also included patients without malignancy. The most frequent type of cancer was gynecologic, followed by colorectal, upper gastrointestinal, lung, genitourinary, hematologic, and breast cancer. More than 50% of patients in the CATCH trial^19^ had metastatic disease, 41.5% in the LITE trial,^20^ and 24.6% in the Romera trial.^21^ The basal distribution of cancer localization and metastasis in the studies that reported the primary cancer localization^19,21^ was similar comparing in tinzaparin and control groups.

A total of 1169 patients with cancer were included in our review from the 3 studies: 900 patients in the CATCH trial,^19^ 200 patients with cancer in the LITE trial,^20^ and 69 patients with cancer in the Romera trial.^21^

These studies assessed 175 IU/kg tinzaparin, once daily by subcutaneous administration. The treatment duration of tinzaparin was 3 months in the LITE trial^20^ and 6 months in both the CATCH trial^19^ and the Romera trial.^21^ Two trials used warfarin as control^19,20^ and the other used acenocoumarol.^21^ There were no major differences between arms in baseline characteristics (Table 1).

**Table 1.** Characteristics of Included Randomized Controlled Trials in Patients With Cancer and Venous Thromboembolism.

	LITE Trial 2006^20^	Romera Trial 2009^21^	CATCH Trial 2015^19^
Number of patients	200	69	900
Sex (% female)	49	45	59
Age, years (mean [SD])	–	62 (15.6)	59 (12.6)
Type of cancer, %	88.5 solid tumors and 11.5 hematologic malignancies	79.8 solid tumors, 7.2 hematologic malignancies, and 13.0 unknown	89.6 solid tumors and 10.4 hematologic malignancies
Metastatic cancer, %	41.5	24.6	54.7
Tinzaparin posology	175 IU/kg, once daily, sc injection for 3 months	175 IU/kg, once daily, sc injection for 6 months	175 IU/kg, once daily, sc injection for 6 months
Vitamin K antagonist posology	Warfarin 5 to 10 mg orally, adjusted daily to maintain the INR between 2 and 3 for 3 months	Acenocoumarol 3 mg orally, adjusted daily to maintain the INR between 2 and 3 for 6 months	Warfarin 1 to 5 mg orally, adjusted daily to maintain the INR between 2 and 3 for 6 months

Abbreviations: INR, international normalized ratio; sc, subcutaneous; SD, standard deviation.

All trials used parallel designs to compare 2 arms,^19–21^ whereas CATCH was a phase 3 trial,^19^ the LITE and Romera studies did not report the phase.^20,21^ One trial was conducted in Asia, Africa, Europe, and North, Central, and South America;^19^ 1 in Canada;^20^ and 1 in Spain.^21^ The reviewed trials included 2,^21^ 30,^20^ and 164 participating^19^ centers. Duration of follow-up varied across trials: 180 days^19^ and 1 year.^20,21^ Whereas the CATCH trial reported protocol registration and was sponsored by a pharmaceutical company (LEO Pharma),^19^ the LITE and Romera trials were investigator-initiated studies that received partial funding/support from LEO Pharma (provision of study drug and drug safety monitoring in the LITE trial; duplex ultrasonography in the Romera) trial.

### Excluded Studies

In total, 27 studies were excluded for the following reasons: they were systematic reviews or meta-analyses; non-systematic reviews; non-RCTs; tinzaparin was not assessed; only short-term assessment of tinzaparin; included patients with cancer but data were unavailable; terminated early; clinical practice guidelines; included few patients with cancer.

### Risk of Bias

**Figure 2.**
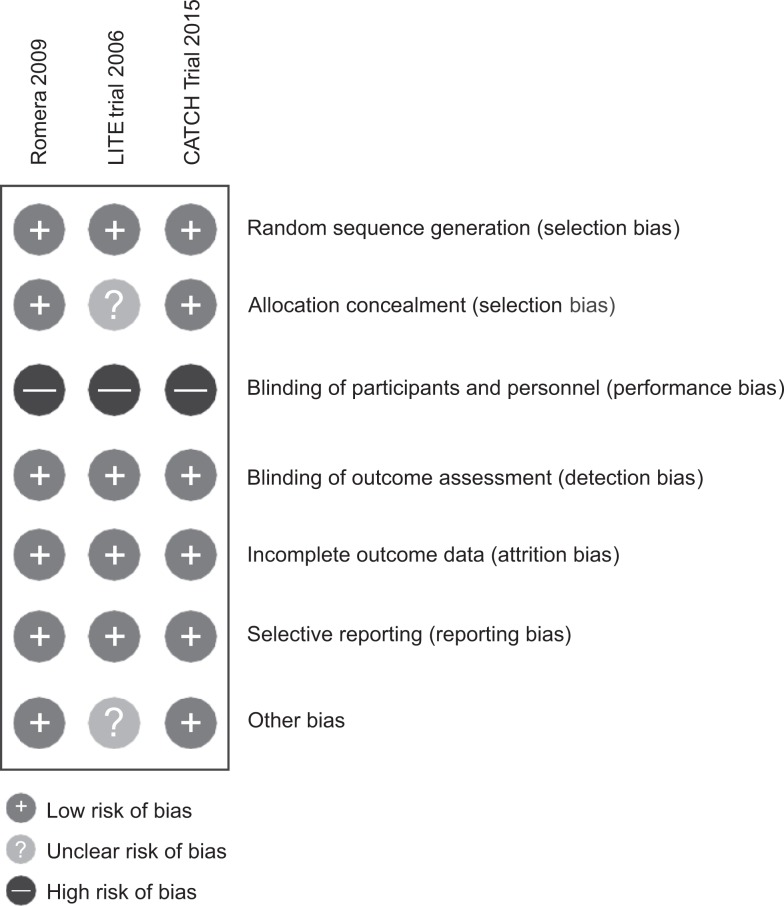
Risk of bias summary: review authors’ judgment about each risk of bias for each included study.

All 3 included trials were rated as having low risk of selection bias regarding random sequence generation (Figure 2).^19–21^

Two trials had low risk of selection bias regarding allocation concealment.^19,21^ One trial reported insufficient information on this item, so it was rated as having unclear risk of bias.^20^

All trials were reported as open label; therefore, they had high risk of performance bias.^19–21^ As such, acknowledgment of the allocated treatment could have influenced compliance with the treatment and the implementation or not of different co-interventions during the study (eg, use of antiplatelets). However, blinding of the interventions is challenging (heparins are administered by fixed dose of subcutaneous injections and antivitamin K is administered orally and dose adjusted by monitoring anticoagulant effect) and could pose ethical concerns.

In the 3 trials, outcome assessment was conducted by reviewers who were not involved in the study conduct and were blinded to the interventions. For this reason, we rated these trials as having low risk of detection bias.

All trials were deemed low risk of attrition and selective reporting bias.^19–21^

The risk of other potential sources of bias was rated low for 2 of the trials^19,21^ and unclear for the LITE trial, due to unreported sample size and inadequate data on the age of participants.^20^

### Primary Outcome

**Figure 3.**
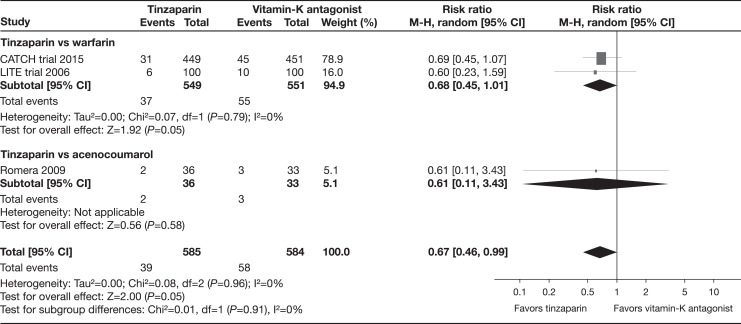
All recurrent venous thromboembolism (subgroup analysis by comparison group).

The results of the meta-analysis are based on 1169 patients with cancer.^19–21^ Pooled data from all 3 trials demonstrated a statistically significant risk reduction (33%) of all recurrent VTE events in participants assigned to tinzaparin compared with those receiving vitamin K antagonist therapy (RR: 0.67, 95% CI: 0.46-0.99, *I* ^2^ = 0%, moderate-quality evidence; Figure 3).^19–21^

The pooled data of CATCH and LITE found no statistically significant difference regarding all recurrent VTE comparing tinzaparin with warfarin (RR: 0.68, 95% CI: 0.45-1.01; *I* ^2^ = 0%).^19,20^ The Romera trial compared tinzaparin with acenocoumarol and the results were not statistically significant for all recurrent VTE events (RR: 0.61, 95% CI: 0.11-3.43).^21^

In the stratified analysis of recurrent VTE by the duration of treatment, results at 3 months were not statistically significant in the single trial providing data (RR: 0.60, 95% CI: 0.23-1.59).^20^ No between-group difference was found between tinzaparin and vitamin K antagonists in the 2 trials with a treatment duration of 6 months (RR: 0.69, 95% CI: 0.45-1.05; *I* ^2^ = 0%).^19,21^ No study had a treatment period longer than 6 months.

**Figure 4.**
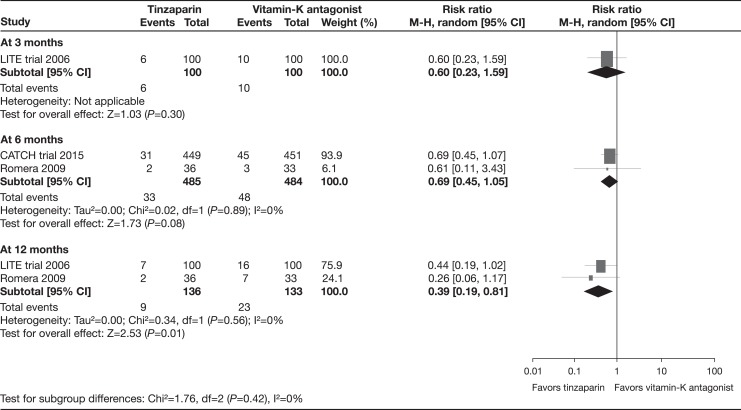
All recurrent venous thromboembolism (subgroup analysis by follow-up time).

In the stratified analysis of recurrent VTE by the length of follow-up, results at 3 months were not statistically significant in the single trial providing data (RR: 0.60, 95% CI: 0.23-1.59).^20^ At 6 months of follow-up, a meta-analysis of the CATCH and Romera trials also showed a non-significant difference of tinzaparin compared with vitamin K antagonists (RR: 0.69, 95% CI: 0.45-1.05; *I* ^2^ = 0%).^19,21^ At 12 months of follow-up, a meta-analysis of the LITE and Romera trials found a statistically significant decrease of all recurrent VTE events in participants receiving tinzaparin versus vitamin K antagonists (RR: 0.39, 95% CI: 0.19-0.81; *I* ^2^ = 0%; Figure 4).^20,21^

We did not perform a sensitivity analysis by the risk of bias because all trials had high risk of bias due to their unblinded study design.

In the sensitivity analysis evaluating only studies that published per protocol data, 1 trial was included.^19^ There was no statistically significant difference regarding all recurrent VTE events comparing tinzaparin to vitamin K antagonist therapy (RR: 0.65, 95% CI: 0.41-1.03).^19^

### Secondary Outcomes

Pooled data from all included trials did not demonstrate any difference between tinzaparin and vitamin K antagonist therapy in all-cause mortality at the end of treatment period (RR: 1.09, 95% CI: 0.91-1.30; *I* ^2^ = 0%, moderate-quality evidence).^19–21^ Similarly, no between-group difference was found in all-cause mortality at the longest follow-up (RR: 1.06, 95% CI: 0.91-1.25; *I* ^2^ = 0%).^19–21^

The pooled data of 3 trials found a significant risk reduction (42%) of all recurrent VTE events at the longest follow-up in participants assigned to tinzaparin compared with those receiving vitamin K antagonist therapy (RR: 0.58, 95% CI: 0.39-0.88; *I* ^2^ = 6%).^19–21^

**Figure 5.**
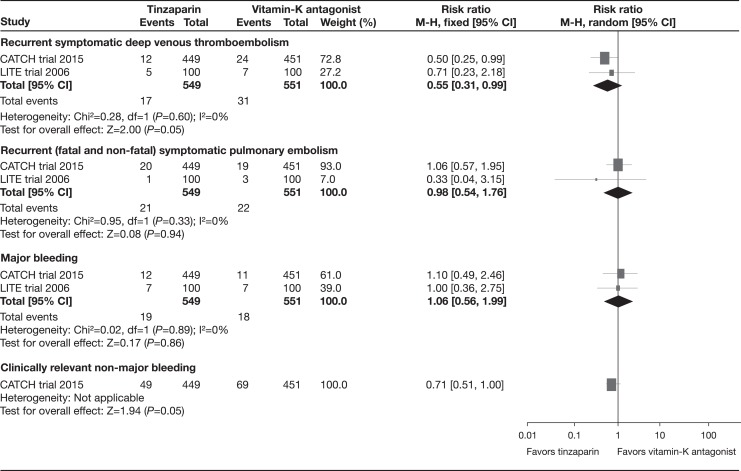
Other secondary outcomes at the end of treatment.

The pooled data of the CATCH and LITE trials found a statistically significant risk reduction (45%) of recurrent symptomatic VTE at the end of the treatment in participants assigned to tinzaparin compared with those receiving vitamin K antagonist therapy (RR: 0.55, 95% CI: 0.31-0.99; *I* ^2^ = 0%, moderate-quality evidence; Figure 5).^19,20^ At the longest follow-up, the pooled results suggested a decrease in recurrent symptomatic VTE events with tinzaparin compared with those receiving vitamin K antagonist therapy (RR: 0.57, 95% CI: 0.32-1.00; *I* ^2^ = 0%).^19,20^ However, the pooled data of the CATCH and LITE trials found no statistically significant reduction in recurrent (fatal and non-fatal) symptomatic PE at the end of treatment with tinzaparin versus vitamin K antagonists (RR: 0.98, 95% CI: 0.54-1.76; *I* ^2^ = 0%, moderate-quality evidence).^19,20^ At the longest follow-up, results were also not significant (RR: 0.46, 95% CI: 0.06-3.70; *I* ^2^ = 75%).^19,20^

In terms of recurrent incidental VTE, the CATCH trial did not find differences in the effect of tinzaparin compared with vitamin K antagonist therapy at the end of the treatment (RR: 0.33, 95% CI: 0.01-8.20).^19^ At the longest follow-up, results were also not significant (0 of 449 [0%] vs 1 of 451 [0.22%]; RR: 0.33, 95% CI: 0.01-8.20).^19^ Furthermore, regarding recurrent incidental PE, no significant differences were found comparing tinzaparin with vitamin K antagonist therapy at the end of the treatment (RR: 0.33, 95% CI: 0.01-8.20).^19^ At the longest follow-up, the results also showed no significant difference (RR: 0.33, 95% CI: 0.01-8.20).^19^

The pooled data of the CATCH and LITE trials found no differences on fatal and non-fatal major bleeding at the end of the treatment between tinzaparin and vitamin K antagonist therapy (RR: 1.06, 95% CI: 0.56-1.99; *I* ^2^ = 0%, moderate-quality evidence).^19,20^

In the CATCH trial that assessed clinically relevant non-major bleeding at the end of treatment, the tinzaparin arm reported lower frequency of this event than the vitamin K antagonist therapy arm (RR: 0.71, 95% CI: 0.51-1.00, moderate-quality evidence).^19^ One trial found no differences between tinzaparin and vitamin K antagonist therapy regarding minor bleeding at the end of the treatment (RR: 1.18, 95% CI: 0.66-2.11).^20^

None of the trials reported data for trivial bleeding, all AEs in general, and those related with the interventions.

### Quality of the Evidence

The overall quality of evidence was moderate, mainly due to a low number of events in the meta-analysis and a small sample size in the LITE and Romera studies (Table 2).^20,21^

**Table 2.** Summary of Findings of Tinzaparin Compared with Vitamin K Antagonist Therapy to Prevent VTE in Cancer at the End of Treatment.^a^

	Comparative Risks^b^		Relative Effect (95% CI)	Participants, n (studies, n)	Quality of the Evidence (GRADE)
	Assumed Risk	Corresponding Risk			
Outcomes Follow-Up: 3 to 6 Months	Vitamin K Antagonist	Tinzaparin (95% CI)			
All recurrent VTE	99 per 1000	67 per 1000 (46-98)	RR: 0.67 (0.46-0.99)	1169 (3)	⊕⊕⊕⊖ Moderate^c^
All-cause mortality	272 per 1000	297 per 1000 (248-354)	RR: 1.09 (0.91-1.30)	1169 (3)	⊕⊕⊕⊖ Moderate^d^
Major bleeding	33 per 1000	35 per 1000 (18-65)	RR: 1.06 (0.56-1.99)	1100 (2)	⊕⊕⊕⊖ Moderate^e^
Clinically relevant non-major bleeding	153 per 1000	109 per 1000 (78-153)	RR: 0.71 (0.51-1.00)	900 (1)	⊕⊕⊕⊖ Moderate^e^
Recurrent symptomatic deep VTE	56 per 1000	31 per 1000 (17-56)	RR: 0.55 (0.31-0.99)	1100 (2)	⊕⊕⊕⊖ Moderate^c^
Recurrent symptomatic PE	40 per 1000	39 per 1000 (22-70)	RR: 0.98 (0.54-1.76)	1100 (2)	⊕⊕⊕⊖ Moderate^e^

Abbreviations: CI, confidence interval; PE, pulmonary embolism; RR, relative ratio; VTE, venous thromboembolism.

^a^Patients with recurrent VTE in cancer; settings: hospital/ambulatory; intervention: tinzaparin; comparison: vitamin K antagonist.

^b^The corresponding risk (and its 95% CI) is based on the assumed risk in the comparison group and the relative effect of the intervention (and its 95% CI).

^c^Downgraded 1 level due to imprecision (low number of events).

^d^Downgraded 1 level due to imprecision (CI overlaps, no effect; cannot exclude important benefit or important harm).

^e^Downgraded 1 level due to imprecision (low number of events and CI overlaps, no effect; cannot exclude important benefit or important harm).

## Discussion

This systematic review of tinzaparin for long-term treatment of VTE in patients with cancer identified 3 RCTs that included 1169 patients with different types of cancer. The percentage of patients with metastatic disease varied from 24% to 54% in the studies.^19–21^ The trials compared 175 IU/kg tinzaparin administered once daily by subcutaneous injection with vitamin K antagonist therapy. Two clinical trials assessed warfarin^19,20^ and 1 assessed acenocoumarol.^21^

This review found evidence of moderate quality suggesting that tinzaparin is associated with a risk reduction of all recurrent symptomatic VTE; and that tinzaparin and vitamin K antagonist therapy have a similar effect on all-cause mortality, on major (fatal and non-fatal) bleeding, clinically relevant nonmajor bleeding, minor bleeding, and recurrent symptomatic PE. None of the trials evaluated “any AEs” or “trivial bleeding” as outcomes.

Of note, the difference in all recurrent symptomatic VTE was driven by a significant reduction in the risk of recurrent DVT; there was no between-treatment difference in the risk of recurrent symptomatic PE. When we stratified the analyses by the time of follow-up, VTE recurrences were significant at 12 months of follow-up but not at 3 or 6 months. Caution is necessary in interpreting the results since they are based on a low number of events because the largest included trial^19^ did not include 12-month follow-up.

Our results are similar to those of a Cochrane review that focused on patients with cancer but included different LMWHs.^8^ The reviews by Akl et al^8^ and Laporte et al^11^ assessed LMWH versus vitamin K antagonists in different participants with and without cancer; this, however, did not include the recently published CATCH 2015 trial. Furthermore, our review focused on tinzaparin and studies in patients with cancer and has overall better quality of evidence (moderate) than the Akl et al’s 2014 review^22^ (from low to moderate), which was downgraded by imprecision and indirectness.

Despite methodological differences with our review, results from Laporte et al^11^ are similar regarding safety outcomes. Laporte et al also showed that tinzaparin compared with vitamin K antagonist significantly reduced the risk of all recurrent thromboembolic events but only at 12 months of follow-up. In our review, we showed that tinzaparin versus vitamin K antagonist significantly reduced the risk of all recurrent thromboembolism events after 3 to 6 months of treatment and at 12 months of follow-up.

We identified some limitations in our systematic review. First, all included trials had high risk of performance bias due to the fact that both patients and researchers were unblinded. However, considering that the outcomes were relevant, objective, measured by diagnostic tests, and that the outcome assessor was blinded, we rated the overall risk of bias as low. Furthermore, concealing interventions (oral tablets vs subcutaneous injections) is difficult to achieve and could pose ethical concerns, particularly in patients with cancer.

Moreover, our results are limited by the primary studies. While adherence could be an important issue in an injectable medication such as tinzaparin, unlike trials observing the use of LMWH following surgery in patients with cancer,^23^ none of the included trials reported on the adherence of the patients to assessed treatments. Furthermore, the number of events in all meta-analyses we performed was low. Moreover, LITE^20^ and Romera^21^ studies had a small sample size and our results were mainly driven by the CATCH trial,^19^ which represented 77.2% of the overall systematic review population. For this reason, the quality of the available evidence was deemed moderate for all included outcomes.

The strength of this review was that the results show consistency and are based on a broad range of types of cancer. A wide search strategy in different databases was implemented, therefore detection bias is unlikely. Only studies comparing tinzaparin with a vitamin K antagonist were identified, and it could be interesting to conduct RCTs comparing the efficacy and safety of tinzaparin with the new oral anticoagulants in the future.

## Conclusion

Our systematic review demonstrated a reduction in the risk of all recurrent thromboembolism in patients with cancer-associated thrombosis managed with long-term tinzaparin compared with vitamin K antagonist therapy. However, according to GRADE methodology, the quality of the available evidence was deemed moderate; this suggests the need for more confirmatory trials, especially with a longer follow-up.
